# Cardiac autonomic modulation of adolescents with different levels of sleep quality

**DOI:** 10.5935/1984-0063.20200027

**Published:** 2020

**Authors:** Carlos Alberto Alves Dias Filho, Carlos José Dias, Rodrigo Barroso, Antonio Carlos Silva Filho, Nivaldo de Jesus Soares Júnior, Andressa Coelho Ferreira, Sara Raquel Dutra Macedo, Janaína de Oliveira Brito Mozani, Bruno Rodrigues, Cristiano Teixeira Mostarda

**Affiliations:** 1 Universidade Federal do Maranhão - Rede Nordeste de Biotecnologia - Laboratório de Adaptações Cardiovasculares ao Exercício, Programa de Pós-graduação - São Luís - Maranhão - Brazil.; 2 Universidade Federal do Maranhão - Programa de Pós-graduação em Saúde do Adulto - Laboratório de Adaptações Cardiovasculares ao Exercício - São Luís - Maranhão - Brazil.; 3 Faculty of Physical Education, Universidade Estadual de Campinas, Educação Física - Campinas - São Paulo - Brazil.; 4 Universidade Federal do Maranhão - Programa de Pós-graduação em Saúde do Adulto - Rede Nordeste de Biotecnologia - Laboratório de Adaptações Cardiovasculares ao Exercício, Educação física - São Luís - Maranhão - Brazil.

**Keywords:** Hypertension, Child, Stress, Psychological

## Abstract

Evaluate the cardiac autonomic modulation of adolescents with different levels of sleep quality.

**Methods:**

Adolescents aged 11 to 17 years who presented themselves an explanation of the project with consent form signed by participated in the study. Anthropometric and hemodynamic data collected, and questionnaires.

**Results:**

Analysis of the variables, age, systolic blood pressure, diastolic blood pressure, waist circumference, body fat and body mass index among the groups with good and poor sleep quality did not present a signiﬁcant difference (p>0.05) in any paired characteristics. Time domain analysis indicated lower values in the vagal modulation with poor sleep quality. In the frequency domain, LF component increased and HF component decreased signiﬁcantly in the group with poor sleep quality. There was also a statistical difference in the LF/HF analysis, the group with poor sleep quality presented an increase in this variable.

**Conclusion:**

The subjects with poor sleep quality present lower cardiac autonomic modulation.

## INTRODUCTION

Cardiovascular diseases are the most important cause of mortality in the world according to the most recent report^1^. In this sense, many studies confirming that the primary risk factors for cardiovascular disease are cigarette smoking, hypertension, elevated serum cholesterol level, sedentary lifestyle and diabetes mellitus (DM)^2^.

Additionally, several studies have identified an increased risk for cardiovascular diseases in individuals who have sleep deprivation, and that this problem can be related to a malfunction of the cardiovascular autonomic modulation. In an exploration of the acute effects of sleep deprivation on the autonomic nervous system in healthy youngsters was observed that, after sleep deprivation, there was a reduction of autonomic modulation during the day, indicating stress of the autonomic system and increased cardiovascular risk^3^.

Recent research has demonstrated that insufficient sleep is a risk factor for hypertension in adults^4,5^. Although the cause is unclear, experimental studies indicate that a shorter sleep period can result in metabolic and endocrine dysfunction, which may contribute to a higher risk of cardiovascular disease^6-8^. According to a study performed by Glos et al.^3^, one night of total sleep deprivation promotes an increase of the sympathetic tone in the early-morning hours. Thus, a change of the sympathetic tone also occurs during subsequent nighttime sleep^3^. A systematic review has shown that heart rate and blood pressure remain high when the sleep recovery is insufficient. Besides, high indexes of sympathetic autonomic modulation and higher blood pressure may predispose to cardiovascular impairment^9^.

Sleep quality and sleep duration may be seen as two different sleep domains, which are associated with the risk of cardiovascular disease. In this sense, sleep quality refers to the subjective indices of how sleep is experienced, including the feeling of being rested when waking up and satisfaction with sleep^10^. On the other hand, sleep duration is a more objective sleep domain, namely the actual time during which the individual is asleep^11^.

Research suggests that sleep deprivation may increase the incidence of cardiovascular events. Thus, a reduction in sleep time has been associated with a variety of problems such as hypertension, myocardial infarction, stroke and stress^12-17^. Sleep deprivation also causes mental stress, as well as myocardial ischemia that when prolonged, may stimulate the development of atherosclerotic lesions^18^.

A previous study found an association between sleep disturbance and cardiovascular risk in adolescents, as determined by high cholesterol levels, increased BMI and hypertension. Since sleep disturbance is highly prevalent in adolescence, these findings demonstrate that cardiovascular disease risk factors track from childhood into adulthood^9,19^. Thus, increase the risks these adolescents acquire cardiovascular diseases in adulthood. Given this, the relationship between sleep deprivation and cardiovascular risk in adolescents is well known. However, few studies evaluated the impact of this variable on autonomic modulation in adolescents. Thus, this study aimed to evaluate cardiac autonomic modulation and risk cardiovascular in adolescents with different levels of sleep quality.

## MATERIAL AND METHODS

### Sample

This is an analytic and transversal study, which included 133 children and adolescents of both the genders, aged between 11 to 17 years, from a single state public school. Students of the selected age group were invited to participate in the study, upon furnishing the informed consent form (ICF) signed by the parents or guardians, which authorized their participation. This study followed Resolution no. 196/96 of the National Health Council of the Brazilian Ministry of Health and was approved by the Permanent Ethics Committee in Research Involving Human Beings of the Federal University of Maranhão (no. 2.673.791).

The students were divided into the following groups: morning class students with good sleep quality - MGSQ; afternoon class students with good sleep quality - AGSQ; and morning class students with poor sleep quality - MPSQ.

The experiment took place as follows: the data were collected during a single session on the scheduled days. The Pittsburg Sleep Quality Index (PSQI) questionnaire and the International Physical Activity Questionnaire (IPAQ) were used to assess the sleep quality and physical activity level, respectively. For the evaluation of sexual maturation, self-assessment was carried out based on the criteria described by Tanner.

Thereafter, the blood pressure of the participants was measured once they had been in a sitting position for 5 min. The electrocardiogram and body composition were then assessed. After all data collection, the adolescents were divided into a group with good sleep quality and a group with poor sleep quality. Lastly, the data were analyzed and tabulated using the computer program (Statistica^®^ 5.0 software). The methods used for the individual steps are described below.

### Blood pressure measurements

For blood pressure measurements, two automated blood pressure monitors were used (Omron^®^ HEM-711 and Omron^®^ 905)^20^. The protocol for blood pressure measurement followed the norms of the VII Brazilian Hypertension Guideline^21^ and the IV Report on the Diagnosis, Evaluation, and Treatment of Hypertension in Children and Adolescents. An optimal cuff size was used according to the arm size of the participants^22^.

Hypertension was defined as blood pressure more than the 95th percentile for the height and blood pressure, following the norms of the IV Report on the Diagnosis, Evaluation, and Treatment of Hypertension in Children and Adolescents^22^ and the Brazilian Hypertension Guidelines^23^.

### Assessment of the heart rate variability

The heart rate variability was obtained using a 12-lead electrocardiogram device (Micromed Wincardio 600Hz, Brasilia, DF, Brazil), and recorded using the program WinCardio 6.1.1. For this evaluation, the students were instructed to remain at rest in a supine position for 10 min. The indices were evaluated using the Kubios Analysis software HRV version 2.0 (Kubios, Finland) for power spectral analyses of heart rate variability in the time and frequency domains. The HRV measures that were evaluated in the time domain included the variables SDNN, RMSSD, pNN50, SD1, and SD2 by nonlinear analysis, in addition to Total variance. In the frequency domain, the spectrum resulting from Fast Fourier Transform modeling was derived from all the data in a minimum 5-min window from the recorded signal. This includes the entire signal variance, regardless of whether its frequency components appear as specific spectral peaks or as nonpeak broadband powers. The frequency bands used for the spectral analysis were low frequency (LF, 0.04-0.15Hz), high frequency (HF, 0.15-0.4Hz), and autonomic balance (LF/HF), a component proposed as a measure of cardiac sympathovagal balance.

### Body composition analysis

The anthropometric evaluation was performed with the participant in the orthostatic position. The weight was measured using a Balmak digital scale (in kg), while the height was measured using a compact stadiometer, type EST 23 (in mm)^24^. All the measurements were performed by a trained professional according to NHBLI, as described by Gordon et al.^25^. Body fat percentage was determined using Maltron^®^ tetrapolar bioimpedance equipment. This equipment generates a current of 800µA with frequency of 50kHz. Prior to this procedure, the subjects were instructed to remove all metallic objects and not to drink alcohol and/or caffeine in the previous 24 hours, as well as to urinate 30 minutes before the evaluation and not to practice physical activity^26^.

### Pittsburgh Sleep Quality Index (PSQI)

Sleep quality and occurrence of sleep disorders were evaluated using the Pittsburgh Sleep Quality Index (PSQI), as originally defined by Buysse^27^. The PSQI uses seven components: (a) subjective quality of sleep, (b) sleep latency, (c) duration of sleep, (d) habitual sleep efficiency, (e) sleep disorders, (f) use of medication to sleep, and (g) daytime sleepiness and disorders during the day. The score for each component was determined separately, on a scale of 0 to 21 points, where greater the value of the score obtained, worse is the quality of sleep. Score values between 0 and 4 represent good sleep quality, those between 5 and 10 represent poor sleep quality, and those greater than 11 indicate sleep disorders.

### Evaluation of sexual maturation

For the evaluation of sexual maturation, the criteria used by Tanner were adopted. This is a method of self-evaluation using images, which takes into account the development of breasts in girls and that of the penis in boys, as well as hair in both genders.

Subsequently, the individuals were classified in one of the following five stages: 1st stage: indicates that the individual is still in childhood (pre-pubertal); 2nd stage: represents the beginning of maturational period; 3rd and 4th stages: shows the continuity of the maturation process; 5th stage: indicates that the individual is a complete adult.

### Statistical analysis

Initially, the data were submitted to the Kolmogorov-Smirnov normality test, showing a normal distribution pattern. One-way ANOVA and Tuckey post hoc tests were used to evaluate the difference among groups. The analysis of cardiovascular risk and sleep quality were estimated for calculating the odds ratio (OR). Also, a cluster of cardiovascular risk factors in adolescents using HRV variables was performed^28^. PSQI score cut-point of ≥5 was used to differentiate high and low-risk groups for future cardiovascular events. The level of significance was set at *p*<0.05. Results are reported as mean value ± standard deviation. We used the Statistica^®^ 5.0 software for data analysis. To evaluate the association between qualitative variables, the chi-squared test was performed.

## RESULTS

Of the 133 adolescents evaluated in the study, 52 were allocated to group MGSQ (32 male and 20 female), 62 were allocated to group AGSQ (28 male and 34 female) and 19 were allocated to group MPSQ (11 male and 8 female). We did not find afternoon class students with poor sleep quality.

There were no significant differences in the analysis of the variables: age, systolic blood pressure, diastolic blood pressure, waist circumference, fat percentage and body mass index between the groups (Table 1).

**Table 1:** Characteristics of adolescents with good or poor sleep quality.

	MGSQ (n=52)	AGSQ (n=62)	MPSQ (n=19)	
Age (years)	14.59 ± 0.32	14.23±0.13	14.33 ± 0.54	
Systolic blood pressure (mmHg)	111.3 ± 1.74	113 ± 2.21	112.4 ± 5.02	
Diastolic blood pressure (mmHg)	66.65 ± 1.22	65 ± 0.9	65.50 ± 3.19	
Waist circumference (cm)	68.93 ± 1.45	68.32 ± 2.11	68.24 ± 3.42	
Body fat (%)	21.85 ± 1.18	20.32 ± 2.24	19.95 ± 1.71	
Body mass index (kg/m^2^)	20.20 ± 0.58	20.43 ± 0.31	19.94 ± 0.91	
PSQI				
Sleep duration	8.17 ± 0.42	9.57 ± 0.74	6.83 ± 0.84	
Sleep efficiency	92.69 ± 1.74	94.82 ± 3.04	87.65 ± 3.78	
Tanner's sexual Maturation index				X^2^
1	3 (3.23 %)	4 (2.78%)	3 (3.98 %)	
2	5 (2.59 %)	2 (2.23%)	1 (3.19 %)	[3.95]
3	32 (35.56 %)	30 (30.60%)	48 (43.83%)	
4	3 (1.62%)	1 (1.39%)	1 (1.99%)	

MGSQ: Morning shift students with good sleep quality; AGSQ: Afternoon shift students with good sleep quality; MPSQ: Morning shift students with poor sleep quality. Values presented as a mean ± standard error; One-way ANOVA with Tuckey post hoc. *p<0.05 vs. MGSQ group and ^#^*p*<0.05 vs. AGSQ group. To evaluate the association between qualitative variables, the chi-squared test was performed.

As observed in Table 2, the mean RR-interval, RMSSD, pNN50 and SD1 were lower in the group MPSQ when compared to the groups MGSQ and AGSQ. Besides, vagal modulation (%HF) was lower in the group MPSQ when compared to the groups with good sleep quality. However, SD2 value was similar among the groups. The sympathetic modulation (LF%) and sympathovagal balance index (LF/HF) were higher in the group MPSQ when compared to the groups with good sleep quality.

**Table 2:** Analysis of the heart rate variability of adolescents with good and poor sleep quality.

	MGSQ (n=52)	AGSQ (n=62)	MPSQ (n=19)
Time domain			
Mean RR (ms)	794.9 ± 19.72	810 ± 14	678.4 ± 29.91*^#^
SDNN (ms)	48.38 ± 1.92	50 ± 2.01	41.71 ± 3.69
RMSSD (ms)	61.80 ± 3.40	65 ± 1.33	43.41 ± 7.51*^#^
pNN50 (%)	39.05 ± 3.23	47.03 ± 2	22.86 ± 6.64*^#^
Nonlinear indices			
SD1 (ms)	43.50 ± 1.70	43.10 ± 3.8	35.03 ± 2.95*^#^
SD2 (ms)	69.58 ± 2.43	69.03 ± 4.3	67.36 ± 5.32
Frequency domain			
LF (ms^2^)	850.5 ± 80.28	811 ± 74.45	844.7 ± 128.2
HF (ms^2^)	1624 ± 137.3	1325 ± 128	925.1 ± 163.0*
LF (%)	35.91 ± 0.69	40.26±1.0	45.45 ± 3.06*^#^
HF (%)	64.09 ± 0.69	59.74 ± 1.0	54.55 ± 3.06*^#^
LF/HF	0.56 ± 0.04	0.58 ± 0.08	1.08 ± 0.12*^#^

MGSQ: Morning shift students with good sleep quality; AGSQ: Afternoon shift students with good sleep quality; MPSQ: Morning shift students with poor sleep quality. Values presented as a mean ± standard error; One-way ANOVA with Tuckey post hoc.

* *p*<0.05 vs. MGSQ group and

# *p*<0.05 vs. AGSQ group.

In Table 3, in the cardiovascular risk analysis, when associated the sleep conditions and heart rate variability indices, we observed that adolescents with RMSSD index less than 49.60 in addition to poor sleep quality have an increased cardiovascular risk (Odds ration: 2.37) than adolescents with good sleep quality. Besides, as shown in the frequency domain, LF greater than 46.10, HF less than 53.80, LF/HF higher than 0.85 in addition to poor sleep quality have increased cardiovascular risk (Odds ration: 8.33) when compared to adolescents with good sleep quality. Regarding the nonlinear indices, we observed that the parasympathetic modulation lower than 35.10 (SD1) in addition to poor sleep quality also presents an increased cardiovascular risk (Odds ration: 3.45) compared to adolescents with good sleep quality.

**Table 3:** Cut-off points and indicators of detection heart rate variability index in cardiovascular risk factors in adolescents.

	Cut-off	Odds ration	Good Sleep Quality (events/non events)	Poor Sleep Quality (events/non events)	95% IC	p-value
SDNN (ms)	> 63.70	2.16	80/37	14/3	[0.584-7.971]	0.12
RMSSD (ms)	> 49.60	2.37	51/66	11/6	[0.822- 6.846]	0.05
PNN50	< 26.10	3.45	48/69	12/5	[1.141-10.431]	0.01
LF (nu)	< 46.10	8.33	42/75	14/3	[2.26-30.666]	0.00
HF (nu)	> 53.80	8.33	42/75	14/3	[2.265-30.666]	0.00
LF/HF	< 0.85	8.33	42/75	14/3	[2.265-30.666]	0.00
SD1(ms)	< 35.10	3.45	48/69	12/5	[1.141-10.431]	0.01
SD2(ms)	>84.80	1.17	86/31	13/4	[0.355-3.864]	0.40

CI: Confidence interval; SDNN: Standard deviation of all RR intervals; RMSSD: Root mean square of the squared differences between adjacent normal RR intervals; PNN50: Percentage of adjacent intervals over 50ms; LF: Low frequency; HF: High frequency.

**Figure 1:**
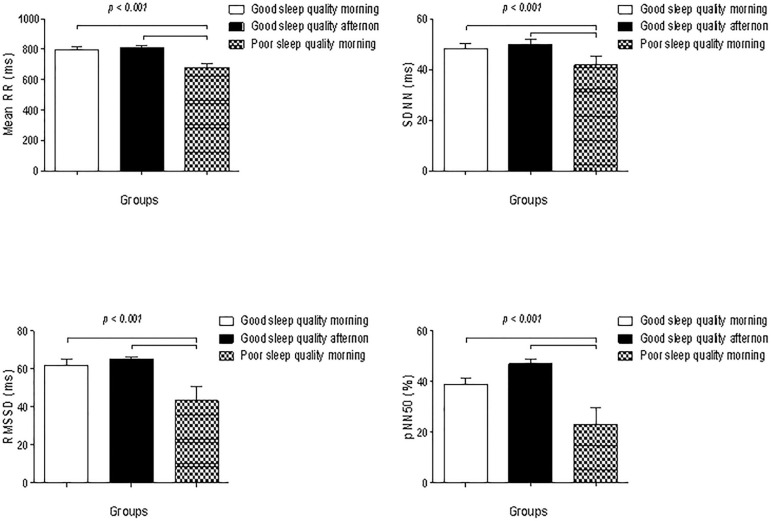
Analysis of the heart rate variability in the domain time. Statistically significant p<0.05. One-way ANOVA; Tuckey post hoc. *p<0.05 vs. MGSQ group and # *p*<0.05 vs. AGSQ group.

In Figure 1, we observed the results of the heart rate variability. The variables mean RR, RMSSD, SDNN and pNN50 were statistically significant when compared to the group MPSQ to the MGSQ and AGSQ groups, observing that the results of these variables were lower in MPSQ group.

## DISCUSSION

This study aimed to evaluate cardiac autonomic modulation and risk cardiovascular in adolescents with different levels of sleep quality. Our results show that poor sleep quality can promotes autonomic dysfunction as observed in the results of the heart rate variability. We highlight the differences observed in the LF/HF ratio, which evidenced this dysfunction in adolescents with poor sleep quality, since several clinical conditions were associated with an increase of morbidity and mortality related to cardiovascular risk factors such as poor sleep quality and insufficient sleep amount^29^.

Thus, the estimates of cardiovascular risk in adolescents were increased when associated with poor sleep quality in addition to heart rate variability indices, evidenced from a reduced parasympathetic modulation (RMSSD, HFnu and SD1) and increased sympathetic modulation (LFnu). In this way, our results show the afternoon shift students that not reported poor sleep quality (AGSQ group) had better cardiac autonomic modulation and better vagal modulation than the MPSQ group.

The results found have significant practical applications. Obtaining cut-points from HRV parameters that discriminate cardiovascular risk in adolescents may help physicians and other health professionals to interpret and staging cardiovascular risk through HRV^28^. Also, it is known that the frequency domain parameters are the best discriminants of cardiovascular risk in adolescents. Thus, the importance of monitoring cardiac autonomic modulation in the first stages of life is highlighted since it has been considered an independent predictor of mortality^30^. Besides, autonomic parameters associated with other cardiovascular risk factors, such as poor sleep quality in adolescents, reinforce the importance of heart rate variability analysis in health parameters and sleep quality in young people.

Adolescent’s health and your sleep patterns are objects of attention in several studies. Previous studies have shown that the PSQI is a valuable tool for assessing subjective sleep quality, being possible to measure sleep quality and estimate the prevalence of poor sleep quality in adolescents. On the other hand, heart rate variability has been used to evaluate sleep problems such as insomnia, sleep-disordered breathing, and periodic limb movements in sleep, besides can use in the screening and monitoring of individuals suffering from these disorders^31^.

In a study conducted by the Healthy Heart Schools’ Program, which aimed to identify adolescents with risk of coronary vascular disease, it was suggested that cardiovascular risk factors are higher in adolescents with sleep disorders^19^ than to adolescents with hypertension, as well as in adolescents with higher body mass index and higher cholesterol levels. These findings are important once sleep disturbance is highly prevalent in adolescence; these findings demonstrate that cardiovascular disease risk factors track from childhood into adulthood^32^. However, in our study there was no correlation between an increase in BMI and blood pressure. Regarding HRV, in previous study performed with children (five to six years) that presented short sleep duration, was observed a decrease in all HRV parameters^33^.

Sleep disorders such as insomnia and sleep-disordered breathing have been associated with an increase of the sympathetic autonomic nervous system in both healthy and no healthy subjects. In this sense, in previous study of our group, we found that sleep quality already influences HRV in childhood. More specifically, poor sleep quality was related to an increase in sympathetic dominance in childhood. Besides, the literature suggests that lack of sleep could have increase sympathetic activity, so promoting metabolic effects including increase in the catecholamines, norepinephrine, and epinephrine levels, through activation of the stress system^9,34^. In this way, sleep quality can affect hormone levels that could have also lead to other changes pathophysiological in these individuals^35^. Thus, it is necessary to have an understanding of the specific pathophysiological pathways linking sleep disorders to several diseases such as hypertension, diabetes and cardiovascular diseases^36^.

In a previous study that evaluated the sleep deprivation, was analyzed the association between stress and body composition for two years (2010 to 2012). In this study, it was observed that short sleep duration, nocturnal awakenings, and lower sleep efficiency may to increase the sympathetic activity^37^.

Additionally, Meier-Ewert^38^ demonstrated changes in baseline blood pressure after partial sleep deprivation. Also, long-term prospective studies have established short sleep duration as a significant risk factor for hypertension and cardiovascular disease, regardless of classic risk factors including obesity and diabetes^39^. This fact suggests that the deleterious effects are proportional and additional to prolonged and repetitive sleep deprivation.

As the sleep deprivation is an increasingly frequent condition from the hectic modern lifestyle, it becomes imperative for clinicians to increase their awareness of gender-based differences in risk factors, lipid profiles, and treatment response to effectively refocus cardiovascular care^40^ and to improve sleep quality in this population. Thus, strategies to improve analysis of the HRV in adolescence may serve for primary prevention of some risk factors for cardiovascular disease in this population.

As discussed earlier, there is evidence that partial sleep deprivation is endemic in our society. Through this study, we were able to demonstrate the clinical importance that partial sleep deprivation promotes once significant changes in the autonomic control are observed. Thus, this study corroborates with the recent epidemiological literature, which suggests that sleep patterns are correlated with morbidity and mortality in cardiovascular disease^39,41^.

It is important to note that our study had some limitations, one of them being the size of the study sample. A greater number of participants in both groups could confirm our results in future studies. Also, for more accurate results, sleep quality can be evaluated by overnight polysomnography (PSG) method.

Therefore, we conclude that adolescents with poor sleep quality present lower cardiac autonomic modulation e increased cardiovascular risk. This may be influenced by the class schedule.
